# Allergies: The Radical Theory of Sneezing

**DOI:** 10.1289/ehp.113-a736b

**Published:** 2005-11

**Authors:** Tina Adler

Anyone with common seasonal allergies knows perfectly well what’s causing their misery: pollen! And allergists know why pollen makes people sneeze: the body’s immune system is releasing a lot of inflammatory cells, including neutrophils and eosinophils, in response to the invading pollen proteins. However, new research reveals that it’s more than just pollen’s proteins wreaking havoc on human airways.

Earlier work had shown that the inflammatory cells the body spews out in response to pollen harbor enzymes called NADPH oxidases. Now researchers report in the August 2005 *Journal of Clinical Investigation* that even before the immune system cranks up, NADPH oxidases in pollen itself generate a type of free radical known as reactive oxygen species (ROS), which interfere with cell signaling pathways and cause the immune system to overreact.

“We demonstrate for the first time to our knowledge that pollen extracts from weeds, trees, and grasses have intrinsic NADPH oxidase activity that induces ROS in airway epithelium within minutes,” the team writes. ROS are formed when NADPH oxidases interact with cells lining the airways. The result is oxidative stress, which health experts suspect exacerbates asthma and allergies. Pollen’s double whammy causes the often quick, intense allergic reaction seen in sensitized patients, explains lead author Istvan Boldogh, a molecular biologist at the University of Texas Medical Branch at Galveston.

The surprising new findings reveal that “pollen is more active than we thought,” says J. David Lambeth, a molecular biologist at Emory University School of Medicine, who wrote a commentary on the study for the same journal. “We knew that pollen can make the body make free radicals, but this study shows that pollen takes an active role in making free radicals itself,” he says.

Plant cells were known to contain NADPH oxidases similar to those found in white blood cells in humans and other mammals. Among other important functions, the oxidases protect the plant against pathogens. However, researchers had not tested pollen for NADPH oxidases, says Boldogh. He and his colleagues uncovered pollen’s double-barreled effect on lungs by exposing sensitized mice to different forms of pollen, some with excess NADPH oxidases added, others that were NADPH-free. When they eliminated the NADPH oxidase activity, the mice had little or no inflammation in their airways and produced few of the cells that indicate an allergic response. When the researchers tested the effects of pollen extracts on cells taken from the lining of the lung, they found that adding NADPH oxidase increased the intracellular levels of free radicals.

Patients may someday use an inhaler containing antioxidants to counter ROS and minimize the effects of pollen, says Boldogh. The team’s recent studies show that a combination of the antioxidants ascorbic acid and *N*-acetyl-l-cysteine prevents airway inflammation in pollen-exposed mice.

But antioxidants available now clear from the lungs too quickly to be effective in people, so companies are looking into developing longer-lasting products, Boldogh says. However, the group warns against developing treatments for patients based on its single study, noting that the results are circumstantial and need to be established in patients, work the team is now attempting.

